# Utilization of photographs taken by citizens for estimating bumblebee distributions

**DOI:** 10.1038/s41598-017-10581-x

**Published:** 2017-09-11

**Authors:** Yukari Suzuki-Ohno, Jun Yokoyama, Tohru Nakashizuka, Masakado Kawata

**Affiliations:** 1 Department of Ecology and Evolutionary Biology, Graduate School of Life Sciences, Tohoku University, 6-3 Aoba, Aramaki-aza, Aoba-ku, Sendai, Miyagi 980-8578 Japan; 2 Department of Biology, Faculty of Science, Yamagata University, 1-4-12 Kojirakawa, Yamagata-shi, Yamagata, 990-8560 Japan; 3 Institute of Regional Innovation, Yamagata University, Yujiri 19-5, Kanakame, Kaminoyama, Yamagata, 999-3101 Japan; 4 Research Institute for Humanity and Nature, Kamigamo-Motoyama 457-4, Kita-ku, Kyoto, 603-8047 Japan

## Abstract

Citizen science is a powerful tool for collecting large volumes of observational data on various species. These data are used to estimate distributions using environmental factors with Species Distribution Models (SDM). However, if citizens are inexperienced in recognizing organisms, they may report different species as the subject species. Here we show nation-wide bumblebee distributions using photographs taken by citizens in our project, and estimated distributions for six bumblebee species using land use, climate, and altitude data with SDM. We identified species from photographic images, and took their locations from GPS data of photographs or the text in e-mails. When we compared our data with conventional data for specimens in the Global Biodiversity Information Facility (GBIF), we found that the volume and the number of species were larger, and the bias of spatial range was lower, than those of GBIF. Our estimated distributions were more consistent with bumblebee distributions reported in previous studies than with those of GBIF. Our method was effective for collecting distribution data, and estimating distributions with SDM. The estimated SDM allows us to predict the previous and future species distributions, and to develop conservation policies taking account of future city planning and/or global climate changes.

## Introduction

Species/biodiversity distributions are fundamental information in ecology, and such data sets have the potential to be “big data.” In recent decades, citizen science programs have attracted much attention from scientists and officials who monitor species/biodiversity distribution^1, 2^. Citizen science monitoring can help to collect large volumes of species’ observation data for various species over a wide range of an entire country. These distribution data have been used for scientific researches about estimates of distributions^3, 4^, changes of distributions^5, 6^, migration^7^, and invasion^8^.

Citizen science monitoring is powerful, but it may have a risk of low quality data when it depends on eyewitness reports. If citizens are inexperienced in recognizing organisms, they may report different species as the subject species. Even if they are familiar with a subject species, identifying species correctly can be difficult task for non-experts due to the confusion and/or the unfamiliarity of related species^9, 10^ and the co-occurrence of mimetic species^11^. In addition, the accuracy of observation points by self-report might be low due to some problems of labor, privacy, and human error.

**Figure 1:**
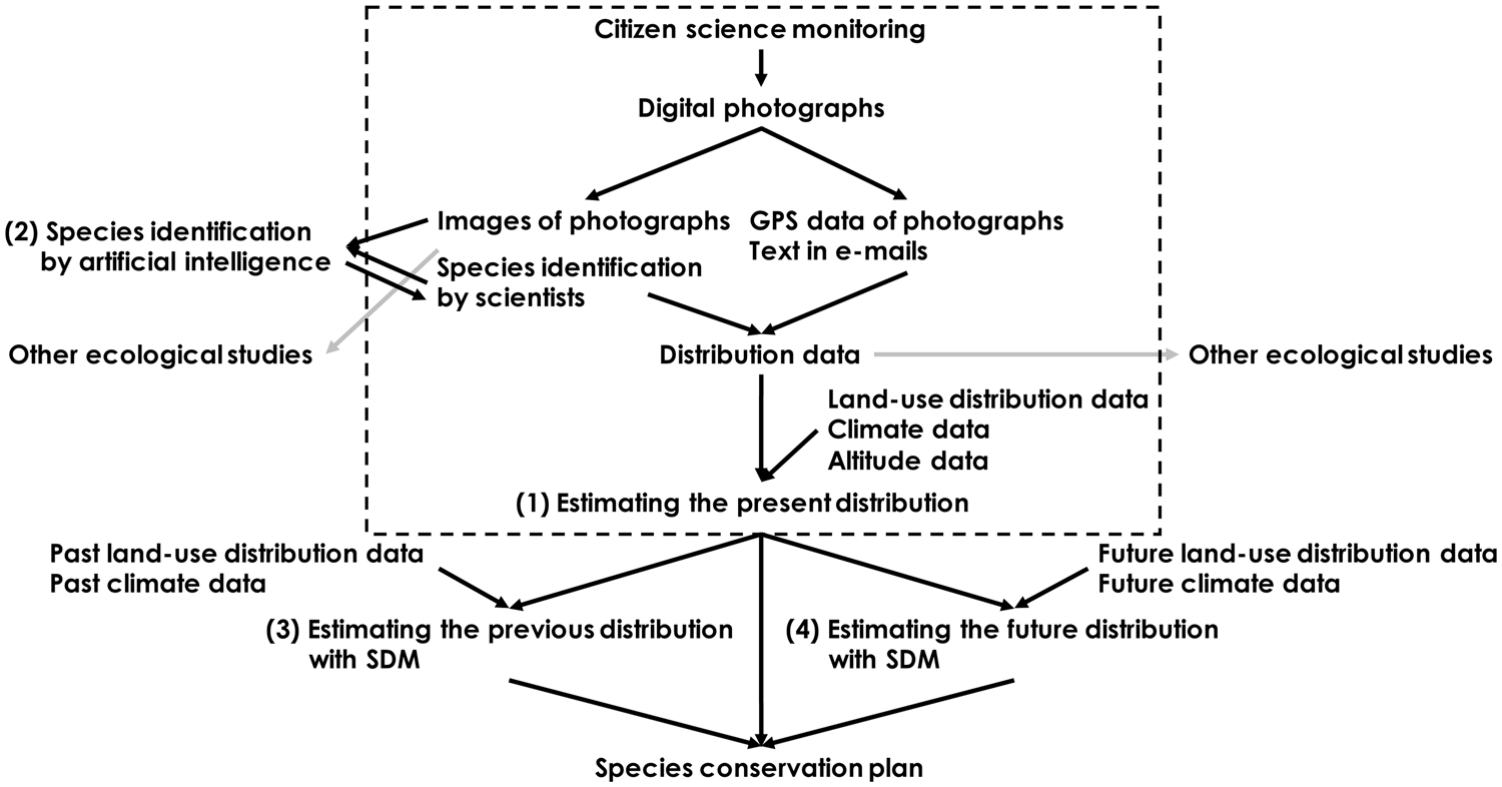
Our method of collecting accurate distribution data in a citizen science program, utilizing these data for scientific researches, and developing species conservation plans and policies. The area enclosed by dashed line indicates this study.

Scientific researches of species/biodiversity distributions require accurate data, especially if species of interest have similar related species and/or mimetic species, and they show small home ranges, and/or low dispersal abilities. We considered the method of collecting accurate distribution data in a citizen science program, utilizing these data for scientific researches, and developing species conservation plans and policies (Fig. 1). We focused on geotagged photographs to collect distribution data because we can identify species correctly using photographic images if they figure characters necessary for identification, and extract correct location of a species’ observation from GPS data in Exif information. At present, many citizen science programs use digital photographs, e.g., iSpot (http://www.ispotnature.org), iNaturalist (http://www.inaturalist.org), iRecord (http://www.brc.ac.uk/irecord), Lost Ladybug Project^12^ (http://www.lostladybug.org), and Bumble Bee Watch (http://bumblebeewatch.org). Species Distribution Models (SDM) can estimate species distributions in the regions where no distribution data are reported, and reduce the effect of a sampling bias that frequently occurred in citizen science programs by spatial filtering and background manipulation^13, 14^. SDM can also estimate the effects of environmental factors on the distributions, and predict the previous/future distributions using the past/future environmental data.

We selected bumblebees (*Bombus* spp.) as subject species, and started our citizen science monitoring program called *Hanamaru-Maruhana* national census (“Bumblebee national census” in English). Bumblebees are ecologically important insects as effective pollinators of both wild and cultivated plants, and they have declined in Europe^15, 16^ and North America^17^. International Union for Conservation of Nature (IUCN) Bumblebee Specialist Group started Red List Assessments of Bumblebees in 2011, and reported that one-third and one-fourth of species in the European and North American bumblebee fauna, respectively, are classified into one of categories of near threatened, vulnerable, endangered, and critically endangered species^18^. Thus, it is urgently necessary to investigate the distributions of bumblebees worldwide. Citizen science monitoring is the most effective method to investigate bumblebee distributions because we can collect the present observation data for multiple bumblebee species over a wide range of an entire country. To estimate bumblebee distributions from citizen science monitoring data, we must obtain accurate bumblebee distribution data. However, it is sometimes quite difficult for citizens because bumblebees involve similar related species and mimicry hoverfly species in the same areas. In addition, they show relatively small foraging range^19, 20^, and their abundance tends to be affected by only neighboring land use (e.g., the percentage of semi-natural habitats within a circle of 750 m radius^21^). Utilization of geotagged photographs will allow us to estimate bumblebee distributions taking the effects of land use into account if we can identify species and locations correctly from photographic images and GPS data in Exif information, respectively.

In this study, we focused on the first step of our method (area enclosed by dashed line in Fig. 1). We investigated bumblebee distribution data using photographs and estimated their distributions using land use, climate, and altitude data with SDM. We collected geotagged photographs of bumblebees by the citizen science monitoring in the “*Hanamaru-Maruhana* national census” bumblebee project from 2013 to 2015 in Japan, and estimated distributions at 1-km resolution for the major six bumblebee species, *Bombus diversus*, *B. ardens*, *B. hypocrita*, *B. ignitus*, *B. honshuensis*, and *B. beaticola*, with Maxent using these photographs and environmental data. To quantitatively evaluate the effectiveness of our method based on citizen science, we compared our results with those using conventional specimens’ data of bumblebees in open database Global Biodiversity Information Facility (GBIF http://www.gbif.org) in Japan. Because the volume of distribution data and the bias towards higher populations in lower-altitude regions frequently become problems in distribution data, we checked the volume of distribution data and the biases of spatial and altitude ranges in our data and GBIF data.

## Results

### Collected photographs

We could collect over 4,100 insect photographs taken by citizens from 2006 to 2015 in our project. Some citizens kindly sent old photographs that they took before the program began. As bumblebees were not familiar to Japanese citizens, many citizens sent photographs of carpenter bees, leaf-cutter bees, honey bees, and hoverflies (please note that the photographs of these bees and hoverflies in Table S1 also included some photographs sent by citizens who knew that they were not bumblebees). Photographic images allowed us to discriminate these photographs.

About 60% of all photographs had GPS data, for which error could range from several to tens of meters when satellites were captured. About 15% had no GPS data, but we could obtain the latitude and longitude data of observation sites in the text in e-mails. These latitude and longitude data were investigated using Google Maps (https://www.google.com/maps) by the citizens who took the photographs, and they were consistent with the address of the observation sites when the text also included the address of the observation sites. The remaining photographs had neither GPS data nor latitude and longitude data in the text, but we could obtain the address of the observation sites in the text in e-mails. We converted the address in the text into latitude and longitude data using Google Maps. Most of the addresses were postal addresses, and could be converted them into latitude and longitude data. If Google Maps defined an area instead of point, we used the center of the area if the area was less than 1 km^2^ (i.e., the maximum possible error should be 500 m). We did not use photographs with low accuracy or absence of location information. The number of excluded photographs were less than 70, and the half of the excluded photographs were other bees or hoverflies. As land use within a radius of 750 m can be important for bumblebees, we considered that our data were accurate enough to incorporate the effects of land use into SDM.

**Figure 2:**
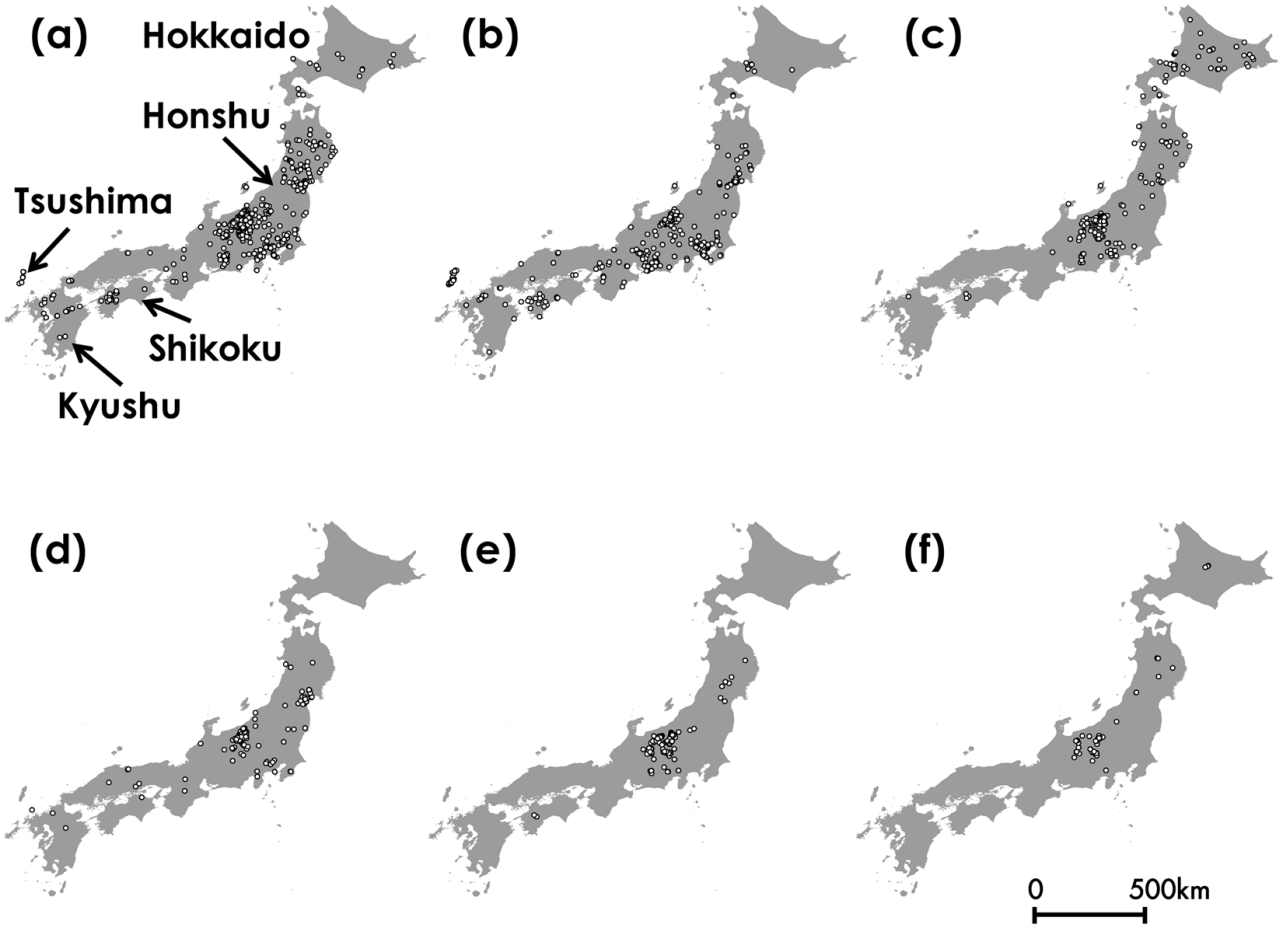
Distributions of six bumblebee species based on our citizen science data. (**a**) *B. diversus*, (**b**) *B. ardens*, (**c**) *B. hypocrita*, (**d**) *B. ignitus*, (**e**) *B. honshuensis*, and (**f**) *B. beaticola*. This map was drawn with the software ArcGIS ver. 10.0 (https://www.arcgis.com/features/index.html).

Among these photographs, 3,185 showed bumblebees (Table S1 in Appendix S1). We identified species and location of 3,143 bumblebee photographs. About 64% of bumblebee photographs had GPS data for observation sites, 15% had latitude and longitude data, and 21% had the address data. Those photographs included 15 species, which are all species inhabit the Japanese archipelago excluding the Kurile Islands with the exception of *B. cryptarum* (Table S2 in Appendix S1). The consistency of bumblebee species identification from photographs was 95% (see Bumblebee species identification in Methods). The numbers of photographs for six major species, which are *B. diversus*, *B. ardens*, *B. hypocrita*, *B. ignitus*, *B. honshuensis* and *B. beaticola*, were high enough to be used for estimating their distributions (902 for *B. diversus*, 821 for *B. ardens*, 369 for *B. hypocrita*, 288 for *B. ignitus*, 207 for *B. honshuensis*, and 145 for *B. beaticola* in Table S1). The locations of the photographs were distributed widely in the whole of the Japanese archipelago (Fig. 2). The consistency of bumblebee species identification for these six species was 97.7% (see Bumblebee species identification in Methods). Therefore, the data for the six species were used for estimating their distributions with Maxent.

In Maxent settings, we can select to remove duplicate presence records in the same cell (about 1 km^2^). After this filtering, we obtained 583 points for *B. diversus*, 411 points for *B. ardens*, 265 points for *B. hypocrita*, 194 points for *B. ignitus*, 140 points for *B. honshuensis*, and 75 points for *B. beaticola* (After RD in Table S1). We extracted presence data from 2013 to 2015, and obtained 563 points for *B. diversus*, 397 points for *B. ardens*, 251 points for *B. hypocrita*, 192 points for *B. ignitus*, 139 points for *B. honshuensis*, and 70 points for *B. beaticola* as the presence data used for Maxent estimates (the number within parentheses in After RD in Table S1).

### Estimation of distributions and the effects of environmental factors on the distributions using our citizen science data

**Figure 3:**
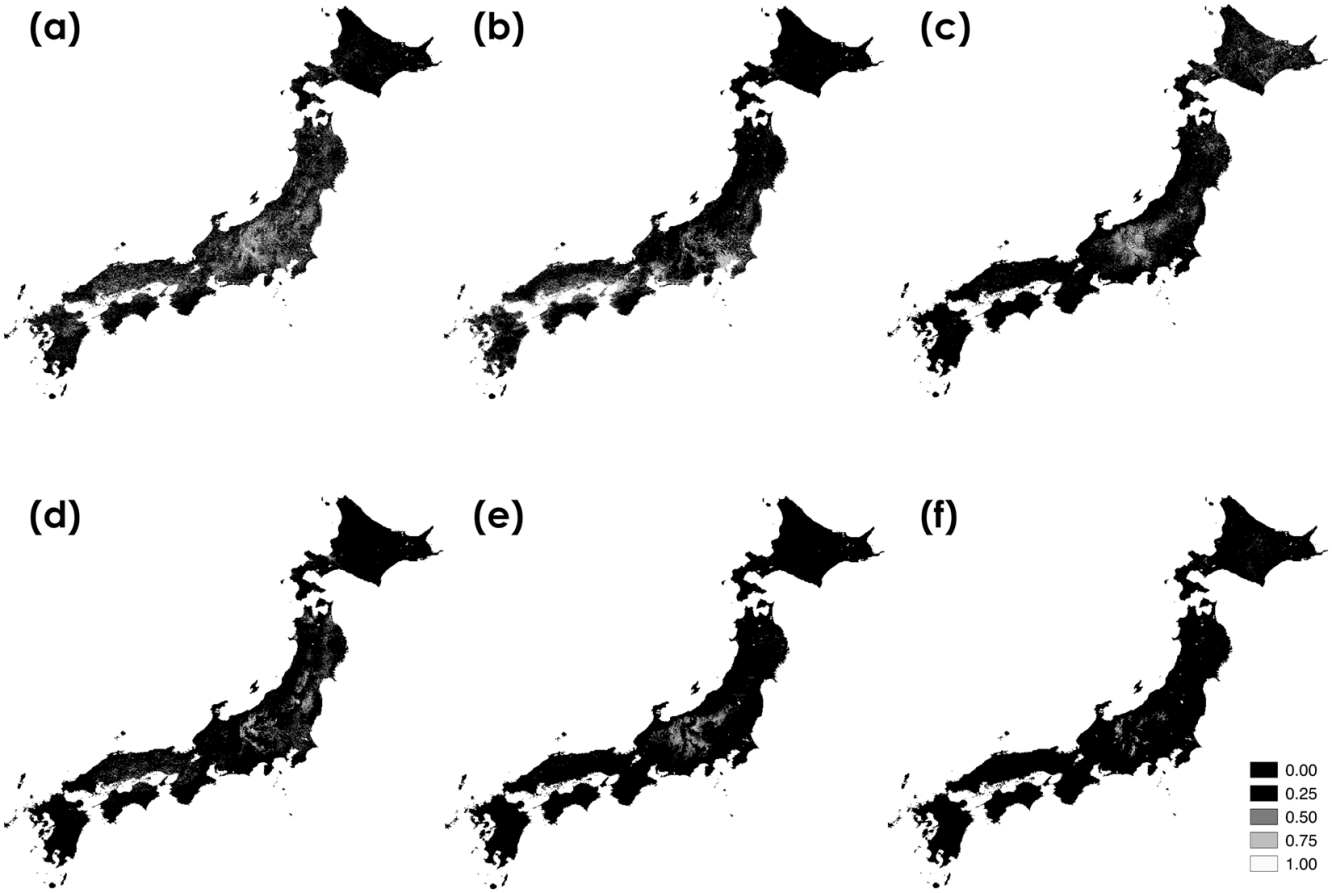
Estimated distributions for six bumblebee species using our citizen science data. (**a**) *B. diversus*, (**b**) *B. ardens*, (**c**) *B. hypocrita*, (**d**) *B. ignitus*, (**e**) *B. honshuensis*, and (**f**) *B. beaticola*. Blighter color indicates higher probability. This map was drawn with the software ArcGIS ver. 10.0 (https://www.arcgis.com/features/index.html).

High-probability areas of distribution for *B. diversus* were the most abundant among the six species (Fig. 3(a)), whereas those for *B. honshuensis* and *B. beaticola* were limited (Fig. 3(e), and (f)). The probabilities for *B. hypocrita* and *B. honshuensis* were high in middle- to high-altitude regions, and that of *B. beaticola* was high in high-altitude regions (e.g., the center of the Japanese archipelago) (Fig. 3(c),(e) and (f)). These estimated distributions corresponded to bumblebee distributions reported in previous studies^22, 23^. AUC was high for all six species (*B. diversus*, mean AUC = 0.778; *B. ardens*, mean AUC = 0.824; *B. hypocrita*, mean AUC = 0.828; *B. ignitus*, mean AUC = 0.905; *B. honshuensis*, mean AUC = 0.930; *B. beaticola*, mean AUC = 0.976).

The distributions for six bumblebee species was determined mainly by forest area, annual mean temperature, and mean altitude (Table 1). Forest areas made the largest contribution to the estimates for *B. diversus* and *B. ignitus*, as well as the second largest for *B. hypocrita*, and the third largest for *B. ardens*. Interestingly, sites with medium forest areas (0.35–0.7×10^6^ m^2^ in 1 km^2^) were suitable for these species (Fig. S1 in Appendix S2 in Supplementary information).

**Table 1:** Environmental factors with average percent contributions greater than 10% in Maxent estimates using our citizen science data. These results were derived from 100 estimates obtained by Maxent.

Species	Environmental factors	Percent contribution	Permutation importance
*B. diversus*	Forest area	30.7	22.6
	Altitude	21.3	13.8
	Temperature	14.5	22.2
*B. ardens*	Land for Building area	45.3	18.5
	Temperature	23.2	42.1
	Forest area	12.7	10.1
*B. hypocrita*	Altitude	32	15.3
	Forest area	21.2	28.9
	Temperature	12.7	13.1
*B. ignitus*	Forest area	39.2	8.8
	Temperature	16.8	33.7
	Precipitation	15.3	16.1
	Altitude	11	5.6
*B. honshuensis*	Altitude	56.8	7.4
*B. beaticola*	Altitude	49.4	11.9
	Temperature	33	81

Annual mean temperature and mean altitude made large contributions to the estimates for five species (Table 1). The suitable temperature and altitude combinations differed among bumblebee species (Appendix S2). These tendencies were also consistent with previous studies^22, 23^. For *B. ignitus*, the percent contributions of annual mean temperature and mean altitude became lower when we took a possible geographical barrier into account (Table S3 in Appendix S3), though the estimated suitable temperature and altitude were almost the same (Fig. S3 in Appendix S3).

### Comparison of our citizen science data with GBIF data

**Figure 4:**
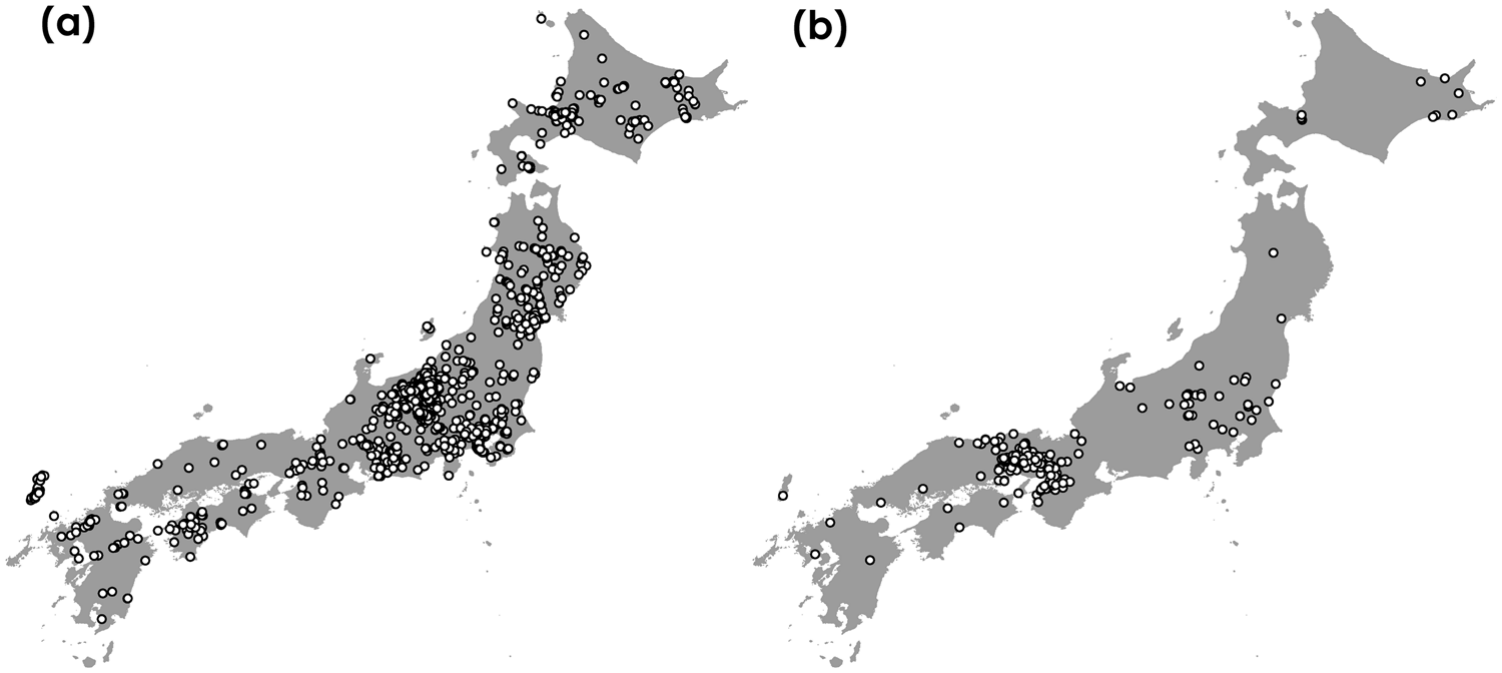
Distributions of all bumblebee species based on our data and GBIF data. (**a**) our citizen science data, and (**b**) GBIF data. This map was drawn with the software ArcGIS ver. 10.0 (https://www.arcgis.com/features/index.html).

As of April 2016, GBIF database contained 1,690 bumblebee records with their locations in Japan, but few were based on recent specimens (e.g., 111 records were from 2006 to 2011, and no records after 2011). Compared with GBIF data, our citizen science data contained about 1.9 times as much as data in GBIF. The number of species identified in our data was also higher than those found in GBIF (n = 15 in citizen science; n = 10 in GBIF). The bias of spatial range in our data was lower than that of GBIF data (Fig. 4). GBIF distribution data were biased toward the western region (Osaka and Hyogo Prefectures) of the Japanese archipelago (Fig. 4(b)), where bumblebees were less frequent than in the northern Japan, because most of GBIF distribution data in Japan depended on the locations of museums that cooperate in uploading the data to GBIF. The altitude range in our data was wider, and mean and median were higher than those in GBIF data (range = 0.2–1904.6 m, mean = 610.9 m, median = 505.8 m in our citizen science data after removing duplicate records; range = 2.3–1792.6 m, mean = 313.3 m, median = 208.9 m in GBIF data after removing duplicate records). When bias^24^ of altitude was calculated as the difference between median values of our/GBIF data and altitude data (median of altitude data = 298.4 m) divided by the range of altitude data (range of altitude data = 0.1–1948.8 m), there was the bias toward higher altitude regions in our citizen science data (bias = 0.106 in citizen science) because some bumblebee species are observed only in higher altitude regions. On the other hand, there was the bias toward lower altitude regions in GBIF data (bias = −0.046 in GBIF).

**Figure 5:**
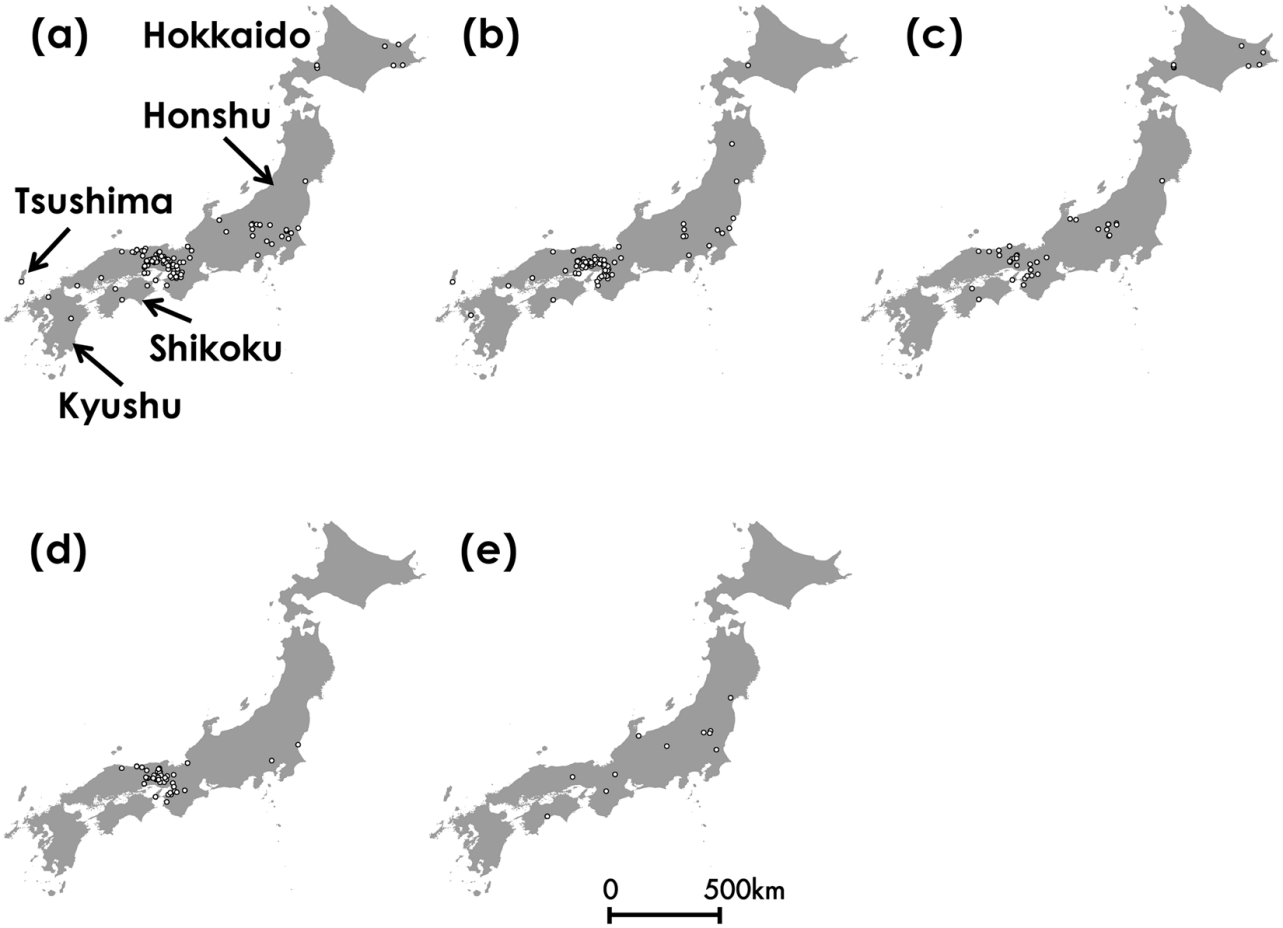
Distributions of five bumblebee species based on GBIF data. (**a**) *B. diversus*, (**b**) *B. ardens*, (**c**) *B. hypocrita*, (**d**) *B. ignitus*, and (**e**) *B. honshuensis*. This map was drawn with the software ArcGIS ver. 10.0 (https://www.arcgis.com/features/index.html).

For six species, the volume of GBIF data was 771 points for *B. diversus*, 527 points for *B. ardens*, 120 points for *B. hypocrita*, 128 points for *B. ignitus*, and 110 points for *B. honshuensis* (Fig. 5). For *B. beaticola*, there were no latitude and longitude data. As we had no records from 2013 to 2015 and a small number of recent records (e.g, 111 records from 2006), we used all records for the six species in the GBIF data. When we removed duplicate presence records within the same cell, this filtering changed the volume of GBIF data from 771 to 122 points for *B. diversus*, from 527 to 83 points for *B. ardens*, from 120 to 43 points for *B. hypocrita*, from 128 to 44 points for *B. ignitus*, and from 110 to 11 points for *B. honshuensis*. It also indicated that GBIF data included many overlapping or biased distribution data.

**Figure 6:**
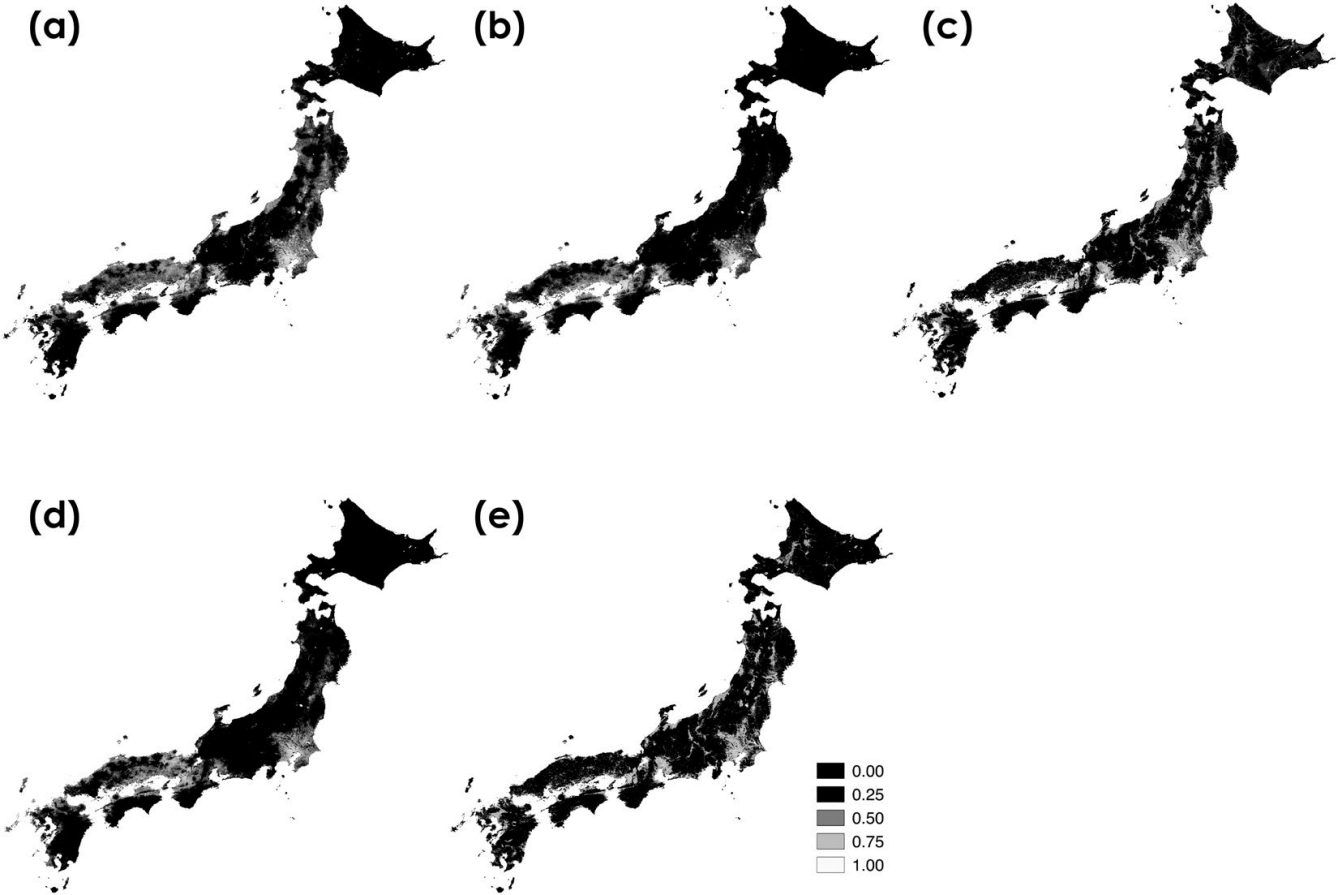
Estimated distributions for five bumblebee species using GBIF data. (**a**) *B. diversus*, (**b**) *B. ardens*, (**c**) *B. hypocrita*, (**d**) *B. ignitus*, and (**e**) *B. honshuensis*. This map was drawn with the software ArcGIS ver. 10.0 (https://www.arcgis.com/features/index.html).

We estimated bumblebee distributions with Maxent using GBIF data. Maxent estimates using GBIF data were greatly different from those using our data (Tables 1 and 2, Figs 3 and 6). High-probability areas were similar among the six species due to higher probabilities at lower-altitude regions (Fig. 6). AUC values for GBIF data was lower than those for our data (*B. diversus*, mean AUC = 0.759; *B. ardens*, mean AUC = 0.785; *B. hypocrita*, mean AUC = 0.712; *B. ignitus*, mean AUC = 0.792; *B. honshuensis*, mean AUC = 0.672), and the standard deviations of ROC were greater than those for our data (Fig. S5). Large contribution of altitude to the estimates was not observed whereas large contribution of land for building area was observed (44.1% for *B. diversus*, 51.2% for *B. hypocrita*, 30.1% for *B. honshuensis* in Table 2). The effects of land use, temperature, and altitude estimated using GBIF data were different from those of our data (Figs﻿. S1, S2, S6 and S7). When we calculated the degree of spatial overlap between binary predictions thresholded by minimum training presence in our data and GBIF data, Cohen’s kappa coefficient of binary predictions indicated fair agreement for *B. iginitus* (κ = 0.28), but poor agreement for the other four species (κ = 0.15 for *B. diversus*, κ = 0.08 for *B. ardens*, κ = 0.03 for *B. hypocrita*, and κ = 0.02 for *B. honshuensis*).

**Table 2:** Environmental factors with average percent contributions greater than 10% in Maxent estimates using GBIF data. These results were derived from 100 estimates obtained by Maxent.

Species	Environmental factors	Percent contribution	Permutation importance
*B. diversus*	Land for Building area	44.1	24
	Temperature	34.4	33.7
*B. ardens*	Temperature	49	61.3
	Land for Building area	34.1	1
*B. hypocrita*	Land for Building area	51.2	29.4
	Forest area	32.7	27.3
*B. ignitus*	Temperature	64	58.1
	Global solar irradiance	12.8	14.1
*B. honshuensis*	Forest area	46.4	65.1
	Land for Building area	30.1	3.7

## Discussion

Our method based on citizen science was effective for investigating country-wide and accurate species distributions (Figs 2 and 4(a)), and estimating the species distributions incorporating the effects of spatial environmental factors into the prediction model (Fig. 3). We collected over 4,100 insect photographs taken by citizens in our citizen science project. Many citizens sent the photographs of other bees as bumblebees, but we could discriminate these data using photographic images. We could collect 3,143 digital photographs of bumblebees taken by citizens from 2006 to 2015. These photographs included 15 bumblebee species present in the Japanese archipelago excluding the Kurile Islands (Table S1). Even the last species we could not obtain data, *B*. *cryptarum*, we could collect the photographs in 2016. The volume and species number of our bumblebee distribution data were larger than those in GBIF, and the bias of spatial range was lower than those in GBIF (Fig. 4). Although distribution data frequently have a sampling bias, estimating the distributions with Maxent using background manipulation by a bias file can reduce the effect of sampling bias^13, 14^. We could estimate six bumblebee distributions at 1-km resolution using land use, climate, altitude data (Table 1 and Fig. 3). These estimated distributions corresponded to bumblebee distributions reported in previous studies^22, 23^. The effects of land use, temperature, and altitude estimated from our data (Table 1, Figs. S1 and S2) were different from those estimated from GBIF data (Table 2, Figs. S6 and S7). When we calculated the degree of spatial overlap of binary predictions in our data and GBIF data, Cohen’s kappa coefficients indicated fair agreement for *B. ignitus* but not for *B. diversus*, *B. ardens*, and *B. hypocrita*, and *B. honshuensis*. It may be attributed to the greater spatial area and altitude ﻿range from which our distribution data were obtained (Figs 2 and 5). In addition, difference in the period for investigation between GBIF records and environmental data may affect the estimates based on GBIF. We concluded that the quantity and quality of our bumblebee distribution data were higher than those in GBIF, but we did not intend to criticize GBIF data for conventional specimens. We hope that museum staffs who have uploaded a large volume of the distribution data to GBIF will be highly valued.

Estimating species distributions using digital photographs with SDM could be a standard method for species conservation planning and policies (Fig. 1). In general, the type of species for which photographs are suitable would be immobile or slow-moving species observed in a low position because they are easier to photograph. Knowing specific locations or situations where the species can be frequently observed will help citizens to take photographs. For example, bumblebees can fly fast, but their movements are slow when they are visiting flowers. We often gave information to citizens about bumblebees’ favorite flowers by direct contacts via e-mails or through our web page. Almost all the bumblebee photographs were of bumblebees collecting nectar and pollen in flowers. Even when species are similar, citizens can take good photographs for identification if they know the characters necessary for identification. We also provide such information in our web page. Interspecific similarity, phenotypic variations, and aging sometimes make it difficult to identify species, but experts who know this information can judge which photographs should be used. It is also effective to collect specimens via citizen science when species identification requires careful observations or dissections. The accuracy of species identification from specimens is higher than that from photographs. However, some organisms are not suitable for collecting by citizens because they are difficult to catch, are rare species, or may hurt citizens (like bumblebees in this study). Furthermore, sending specimens involves more work for citizens than sending photographs via e-mails.

We found that our method had many benefits for us, but it also had several problems. The biggest problem was the cost of identification^10^. In iSpot, crowdsourcing by participants was used to identify the species in photographs^9^. However, it is difficult for ordinary citizens to identify species correctly in photographic images, except for the limited number of species that are familiar to citizens. As scientific research and species conservation require correct scientific names, increasing the accuracy of species identification and reducing the cost of identification are major issues in citizen science monitoring. At present, we are developing a method based on artificial intelligence to identify species in photographic images ((2) in Fig. 1). If artificial intelligence can be used for accurate identification, citizen science monitoring data could be applied more easily to scientific research and species conservation.

The second biggest problem was the uncertainty of species identification from photographic images. We did not have direct evidences that species identification from photographic image was highly accurate, but we could show high consistency of identification of bumblebee species by J. Yokoyama: 95% for 15 bumblebee species, and 97.7% for 6 species used for estimating their distributions. These values were higher than about 85% found in a previous study using bumblebee photographs in two field guides in the UK, although the consistency of the previous study was measured across three trials in the same experiment of the species match-mismatch test^25^. High consistency of species identification in this study would indicate high ability of species identification in our system (combination of trained volunteers and an expert) and difference in bumblebee species between countries. In this study, it was relatively easy for experts to identify Japanese bumblebee species from photographic images because the number of species in this study was only 16 (compared with 64 species in Europe and 46 species in North America), and bumblebee color patterns and body proportions (e.g., head length and length/width ratio) are different among species except for *B. deuteronymus* and *B. pseudobaicalensis*. As it was hard to distinguish these two species only from photographic images, we showed all photographs of these two species in Table S1. There was uncertainty in identification based on photographs due to interspecific similarity, phenotypic variations, and aging (older individuals sometimes lose their hairs and colorations), but the uncertainty can be minimized by looking multiple photographs taken from different angles, and information about visited flowers (e.g., long-tongued bumblebee species visit long-tube flowers), bumblebee behaviors (e.g., nectar robbing), and observation locations (Please note that the consistency of bumblebee species identification from photographic images was measured using one photograph instead of multiple photographs from different angles.) Identification by several experts will increase the accuracy of species identification more.

Our study showed that land use types and their areas greatly affected bumblebee distributions as well as temperature and altitude. The effects of land use areas are usually hard to be detected from low-resolution distribution and environmental data when subject species have small foraging/home ranges^21^ or low dispersal abilities. In our study, we could collect accurate distribution data and estimate distributions at resolution of 1 km. These allowed us to detect the effect of forest area on bumblebee distributions and the forest area was shown be a major contribution to distributions of four of six bumblebee species. Habitat loss and declines in flower resources due to land use changes are considered to be major factors to cause wild bee declines^26, 27^. Cameron *et al*.^17^ estimated the probability of occurrence for bumblebees in North America with Maxent, however; it was estimated using only climate data at the resolution of 10 km. To develop conservation policies of bumblebees, particularly causes of decline of bumblebees are resulted by anthropogenic factors, it is essential to estimate bumblebee distributions taking the effects of land use distribution into account.

The effects of environmental factors on bumblebee distributions estimated using our citizen science data were consistent with bumblebee ecology. Sites with medium forest areas (0.35–0.7 × 10^6^ m^2^ in a 1 km^2^ in Fig. S1 in Appendix S2 in Supplementary information) were suitable for *B. diversus*, *B. ardens*, *B. hypocrita*, and *B. ignitus*. It would be attributed that these species favored forest edges that provide nesting sites inside the forest and foraging sites outside the forest simultaneously^28^. In addition to the preference to forest edges, East Asian traditional agricultural landscape “Satoyama”, which is forest-farmland mosaic landscape maintained by human, could explain why sites with medium forest areas were suitable for above four species (see Ushimaru *et al*. ^29^ for details regarding *B. diversus* and *B. ardens*). Satoyama is considered to play an important role in maintaining biodiversity in Japan^30^. As Satoyama comprises a complex landscape structure, it simultaneously provides nesting sites in forests, and foraging sites at forest edges, grasslands, crop fields, and farmers’ gardens within a small area. The survival and persistence between successive colony cycle stages for bumblebees are strongly related to the area of natural and semi-natural land covers with non-woody, annual or perennial flower mixtures^31^. Satoyama should be an important habitat for *B. diversus*, *B. ardens*, *B. hypocrita*, and *B. ignitus*. The other effects of environmental factors on bumblebee distributions were discussed in Appendix S2. The effects of environmental factors estimated by Maxent were well consistent with bumblebee ecology reported in previous studies^21, 28, 29, 31^, but we must further investigate the effects of environmental factors on bumblebee distribution, because detecting the effects of environmental factors requires greater consideration of alternative models.

Our method could be used for developing species conservation policies. In the case of this study, medium forest areas were important for bumblebees. In order to develop detailed conservation policies, we must specify the locations where high-probability areas have been shrinking. Thus, we will estimate the previous probability for bumblebees using the past climate data and the past distribution of land use types ((3) in Fig. 1). Comparisons of the current and previous probabilities will help to identify areas that require the protection and reconstruction of medium forest areas for promoting the conservation of bumblebees. In addition to land use changes, we must consider the effects of future global warming and climate change on bumblebee species. Among six species, susceptive species to global warming would be *B. hypocrita*, *B. honshuensis*, and *B. beaticola*. *B. hypocrita* and *B. honshuensis* inhabit in medium-high altitude region of medium temperature, and *B. beaticola* inhabit in high altitude region of low temperature in the Japanese archipelago (Appendix S2). Increase of temperature by global warming will cause bumblebee distribution shift to higher altitude regions^32^ or phenology change to earlier season. If distribution shift and phenology change differ from those of their host plants^33^, it may cause the decrease of bumblebee abundances. In addition to their host plants, forest distributions will affect the distributions and abundances of bumblebees, such as *B. diversus*, *B. ardens*, *B. hypocrita*, and *B. ignitus*. To conserve bumblebees, it is essential to consider the changes in host plant and forest distributions as well as the changes in bumblebee distributions themselves and abundances. Thus, we will estimate the future probability for bumblebees using the future climate data and the future distribution of land use types ((4) in Fig. 1).

Except for *B. honshuensis* that is an endemic species of Japan, five species considered in this study, *B. diversus*, *B. ardens*, *B. hypocrita*, *B. ignitus*, and *B. beaticola* widely inhabit in East and/or North Asia. Estimating distributions for five species in East and North Asia based on our distribution data should contribute to bumblebee studies and conservation worldwide.

## Methods

### Bumblebee species identification

Sixteen bumblebee species including one kleptoparasitic species and one exotic species inhabit the Japan archipelago excluding the Kurile Islands (Table S2 in Appendix S1 in Supplementary information). Most of them also inhabit in East and/or North Asia, and seven of these species also inhabit in Europe. Species of bumblebees were identified from photographic images by one of the authors, J. Yokoyama. We tested species identification using 100 bumblebee photographs in which species had been identified two or three years ago and data on the location and date of observation were available. The consistency of bumblebee species identification by J. Yokoyama was 95% for 15 bumblebee species, and 97.7% for 6 species used for estimating their distributions.

### Collection of photographs

Using our web page (http://meme.biology.tohoku.ac.jp/bumblebee/index.html), Facebook (https://www.facebook.com/hanamarumaruhana), and Twitters (https://twitter.com/Hanamaruchan870), we asked citizens to take photographs of bumblebees in nature and submit them to our project. About 100 citizens registered as project members from 2013 to 2015, but many non-registered citizens also submitted photographs to us. Photographs were collected by e-mail or Mobile Phone System and Cloud Services provided by Fujitsu (http://bio.ikimonosirabe.info/psystem). Mobile Phone System and Cloud Services are tools of photograph database developed by Fujitsu, which are provided to citizen science programs as a corporate social responsibility activity. These tools can arrange photographs submitted by e-mail, display the locations where photographs were taken on Google Maps, and output the distribution data as a csv file.

### Estimation of distributions and the effects of environmental factors on the distributions

#### Outline

To estimate bumblebee distributions, we used the species distribution model called Maxent^34–36^. Maxent estimates the distribution based on presence-only data and environmental data. We obtained presence data from GPS data of bumblebee photographs or the text in e-mails. To reduce the sampling bias of the presence data, we applied removing duplicate presence records and background manipulation using a bias file in Maxent. Environmental data including land use, climate, and altitude data were used to estimate bumblebee distributions. In the following, we described the details of presence data and bias file, environmental data, and Maxent settings used in this study.

#### Presence data and bias file

Species of bumblebees were identified from photographic images, and their locations were taken from GPS data in Exif information of photographs or the text in e-mails. We extracted presence data for six species using photographs taken from 2013 to 2015. We could identify subspecies of four species (*B. diversus*, *B. ardens*, *B. hypocrita*, and *B. beaticola*), but we did not discriminate among them because the discrimination of subspecies did not greatly affect the results of Maxent estimates (Appendix S3).

Presence data frequently have a sampling bias, but spatial filtering and background manipulation using a bias file can reduce the sampling bias effect in Maxent^13, 14^. In Maxent settings, we selected “remove duplicate presence records” within the same cell (about 1 km^2^). To secure the volume of presence data in both of our citizen science data and the GBIF data, we did not filter further. Manipulation of the background dataset using a bias file is more effective than spatial filtering when the sample size in insufficient to allow spatial filtering^14^.

Following tutorial of Maxent in International Biological Information System (IBIS) (http://ibis.colostate.edu/cwis438/websites/IBIS/Home.php)^37^, a bias file was prepared using the municipalities map distributed by the Environmental Systems Research Institute (ESRI) and the presence data for all bumblebee species collected from photographs with the software ArcGIS ver. 10.0 (https://www.arcgis.com/features/index.html). The bias file determined the areas where citizens took bumblebee photographs, which were used for background point selection by Maxent. The environmental variables in background points were compared with those in the presence data for the subject bumblebee species. The presence data of other bees (e.g., carpenter bees) were not used to prepare the bias file in this study because some citizens who took only other bees’ photographs might have a low ability to find bumblebees, and they might have been unable to take the photographs of bumblebees even if bumblebees inhabited there. If citizens have good ability to find bumblebees and send absence data, the use of the absence data for the bias file will reduce the risk of ignoring absences in areas where there are truly no bumblebees present.

#### Environmental data

Land use, climate, and altitude data were obtained from National Land Numerical Information download service site (http://nlftp.mlit.go.jp/ksj-e/index.html). The period of land use, climate, and altitude datasets are 2014, average from 1981 to 2010, and 2011, respectively (Table S4). The spatial resolution of all datasets is 1 km, but land use data listed a cover area (m^2^) within 1 km^2^ for each land use type. Land use data was produced using digital maps in the Geospatial Information Authority of Japan and satellite images of SPOT and RapidEye by National Land Numerical Information. Land use data published in 2014 listed the areas of 12 land use types within 1 km^2^, but we eliminated railroad and road areas because they were small areas, and their contributions to the model was very low (0–1%) in the preliminary estimates obtained by Maxent. Climate data listed 84 weather variables, but these variables were strongly correlated with each other. Thus, we selected annual precipitation, annual mean temperature, maximum snowfall, and mean total global solar irradiance (Table S4 in Appendix S4 in Supplementary information), which had correlation coefficients less than 0.75. We also selected mean altitude from nine variables in altitude data. All pairs of selected variables in land use, climate, and altitude data had correlation coefficients less than 0.75. We selected the data of the Japan archipelago excluding the Kurile Islands and the Ryukyu Islands south of Yakushima island with ArcGIS, and converted these shape file data to ascii file data with ArcGIS for Maxent use.

#### Maxent settings

We used the software Maxent ver 3.3.3 k. In Maxent, the Area Under the receiver operating characteristic Curve (AUC) represents the accuracy of Maxent estimate. An AUC value close to 1 means that the accuracy is very high, though AUC by itself can be a misleading measure of predictive performance^38^. Percent contribution represents the relative contribution of each environmental variable to Maxent estimate. Permutation importance represents the relative effect of permuting each environmental variable. High permutation importance means that the accuracy of estimate is reduced seriously by changing the location of the variable. The marginal response curve represents how an environmental variable changes the Maxent prediction with the average sample values of other environmental variables.

The default settings for functional forms are auto features, which can produce complex response curves and they sometimes result in data overfitting. Restricting functional forms is effective for avoiding data overfitting^39, 40^. Therefore, we selected estimates with linear and quadratic features in functional forms. In addition, a large regularization multiplier can reduce data overfitting^41^, but we found no difference between the estimates with regulation multipliers of 1 and 2 (data not shown). Hence, we selected the estimations with the default regularization multiplier of 1. The other settings are random test percentage = 25; maximum number of background points = 10,000; replicates = 100; replicated run type = subsample.

When we calculated Cohen’s kappa coefficient based on the binary prediction areas using our data and GBIF data, we used all presence data, and set as random test percentage = 0; maximum number of background points = 10,000; replicates = 1; and apply threshold rule = minimum training presence.

### Comparison of our citizen science data with those in GBIF

To quantitatively evaluate the effectiveness of our method, we compared our citizen science data with the bumblebee data in Japan registered with GBIF. Bumblebee data in GBIF consist of specimen data in museums in Japan (e.g., Japanese specimen database in S-Net http://science-net.kahaku.go.jp).

We investigated the volume and the species number of bumblebee data in GBIF. The bumblebee data in GBIF were *B. diversus* ^42^, *B. ardens* ^43^, *B. hypocrita* ^44^, *B. ignitus* ^45^, *B. honshuensis* ^46^, *B. ussurensis* ^47^, *B. deuteronymus* ^48^, *B. pseudobaicalensis* ^49^, *B. hypnorum* ^50^, and *B. terrestris* (an exotic species)^51^. We also investigated the volume of presence data of six species in GBIF data after removing duplicate presence records in Maxent settings. We estimated bumblebee distributions using the presence data in GBIF with Maxent. A bias file was made from the presence data of all bumblebee species in GBIF.

### Data availability

At this time, our bumblebee distribution data generated during the current study are not publicly available due to rearranging them for upload to GBIF. The bumblebee distribution data are available from the corresponding authors on reasonable request, and will be available from GBIF.

## Electronic supplementary material

Supplementary information
