# Supplementary material for: Utilization of photographs taken by citizens for estimating bumblebee distributions
Source: Sci Rep. 2017 Sep 11;7:11215. doi: 10.1038/s41598-017-10581-x (PMC5594003; doi:10.1038/s41598-017-10581-x)
Supplement: Supplementary file 1 — Supplementary information [file 41598_2017_10581_MOESM1_ESM.doc]

Utilization of photographs taken by citizens for estimating bumblebee distributions

Yukari Suzuki-Ohno, Jun Yokoyama, Tohru Nakashizuka, and Masakado Kawata

Supplementary Information

Appendix S1.

Table S1. Species and the number of photographs sent by citizens in our citizen science project, and the number of presence data after Removing Duplicate records within the same cell (RD). The number within a parenthesis for six species represents that the number of presence data after RD from 2013 to 2015.

| Scientific name | No. | After RD |
| --- | --- | --- |
| Bumble bees |  |  |
| *Bombus diversus* | 902 | 583 (563) |
| *Bombus ardens* | 821 | 411 (397) |
| *Bombus hypocrita* | 369 | 265 (251) |
| *Bombus ignitus* | 288 | 194 (192) |
| *Bombus honshuensis* | 207 | 140 (139) |
| *Bombus beaticola* | 145 | 75 (70) |
| *Bombus consobrinus* *wittenburgi* | 131 | 35 |
| *Bombus deuteronymus maruhanabachi*^a^ | 74 | 32 |
| *Bombus ussurensis* | 37 | 9 |
| *Bombus hypnorum* | 27 | 12 |
| *Bombus deuteronymus deuteronymus*^a^ and  *B. pseudobaicalensis*^b^ | 26 | 15 |
| *Bombus yezoensis*^c^ | 11 | 5 |
| *Bombus schrencki* | 8 | 7 |
| *Bombus norvegicus* (kleptoparasitic species) | 7 | 1 |
| *Bombus terrestris* (exotic species) | 101 | 61 |
| unidentified (bad photographs and continuous shooting) | 31 | - |
| Carpenter bees |  |  |
| *Xylocopa appendiculata circumvolans* | 318 | 202 |
| *Xylocopa tranquebarorum* (exotic species) | 4 | - |
| Honey bees |  |  |
| *Apis mellifera* (exotic species) | 235 | 141 |
| *Apis cerana japonica* | 101 | 71 |
| Other bees and wasps | 281 | - |
| Others (e.g., hover files) | 68 | - |

^a^ We discriminated the number of photographs for subspecies *B. deuteronymus maruhanabachi* because they are observed in a limited region.

^b^ We described the sum of the photographs of *B. deuteronymus deuteronymus* and *B. pseudobaicalensis* because it was impossible to discriminate them using photograph images alone (their difference is only a bunch of black hair on the abdomen).

^c^ Synonym of *B. consobrinus* in William (2016).

Reference

Williams, P.H. Bombus bumblebees of the world. London: The Natural History Museum. http://www.nhm.ac.uk/research-curation/projects/bombus/index.html (2016)

Table S2. Bumble bee species observed in the Japanese archipelago excluding the Kurile Islands.

| Subgenus | Species and Taxonomic Authority | Distribution |
| --- | --- | --- |
| *Megabombus* | *consobrinus wittenburgi* Vogt | The middle region of Honshu |
|  | *yezoensis*^1^ Matsumura | Hokkaido |
|  | *diversus diversus* Smith | Honshu, Shikoku and Kyusyu |
|  | *diversus tersatus* Smith | Hokkaido |
|  | *ussurensis* Radoszkowski | The middle region of Honshu |
| *Thoracobombus* | *honshuensis* Tkalcu | The southern region of Hokkaido, Honshu, and Shikoku |
|  | *schrencki albidopleuralis* Skorikov | Hokkaido |
|  | *deuteronymus deuteronymus* Shulz | Hokkaido |
|  | *deuteronymus maruhanabachi* Sakagami et Ishikawa | The middle region of Honshu |
|  | *pseudobaicalensis* Vogt | Hokkaido and the northern region of Honshu |
| *Pyrobombus* | *ardens ardens* Smith | Honshu, Shikoku and Kyusyu |
|  | *ardens sakagamii* Tkalcu | Hokkaido |
|  | *ardens tsushimanus* Sakagami et Ishikawa | Tsushima Island |
|  | *hypnorum koropokkrus* Sakagami et Ishikawa | Hokkaido |
|  | *beaticlola beaticola* Tkalcu | High altitude region of Honshu |
|  | *beaticola moshkarareppus* Sakagami et Ishikawa | High altitude region of Hokkaido |
| *Bombus* | *hypocrita hypocrita* Perez | Honshu |
|  | *hypocrita sapporoensis* Cockerell | Hokkaido |
|  | *ignitus* Panfilov | Honshu and Kyushu |
|  | *cryptarum*^2^ Fabricius | The eastern region of Hokkaido |
|  | *terrestris*^3^ Linnaeus | Hokkaido and Honshu |
| *Psithyrus* | *norvegicus japonicus* Yasumatsu | The middle region of Honshu |

1. Synonym of *B. consobrinus* in William (2016).

2. Synonym of *B. florilegus* Panfilov.

3. exotic species.

Appendix S2. The effects of land use types, temperature, and altitude on bumblebee distributions estimated using our citizen science data

The percent contribution of environmental factor indicates the relative importance of the environmental factor for the prediction, and the marginal response curve indicates how the environmental factor increase the prediction with the averaged sample values of the other environmental variables. The percent contributions of forest areas were high for *B. diversus*, *B. ardens*, *B. hypocrita*, and *B. ignitus* (Table 1). The marginal response curve of forest area for these species had peaks at medium forest areas (0.35–0.7×10^6^ m^2^ in 1 km^2^ in Fig. S1(a), (b), (c), and (d)). The percent contribution of land for building area was the largest for *B. ardens* (Table 1). The marginal response curve of land for building for *B. ardens* was an increasing function versus the area of land for building (Fig. S1(e)).

The percent contributions of temperature and altitude were high for five species (Table 1). The marginal response curve of temperature for *B. ardens* was an increasing function versus the increase in the annual mean temperature (Fig. S2(b)). The temperature response curve for *B. diversus* and *B. ignitus* had peaks at relatively high temperatures (11–13°C in Fig. S2(a) and (d)), those for *B. hypocrita* and *B. honshuensis* had peaks at medium temperatures (6°C in Fig. S2(c) and (e)), and that for *B. beaticola* had a peak of low temperature (0°C in Fig. S2(f)). At low altitude, the marginal response curves of altitude for *B. diversus*, *B. ardens*, *B. hypocrita*, and *B. ignitus* kept at a specific level (0.5–0.7 at 0 m in Fig. S2(g), (h) (i) and (j)) whereas those for *B. honshuensis* and *B. beaticola* were very low (0–0.2 at 0 m in Fig. S2(k) and (l)).

The effects of environmental factors on *B. ardens* distribution were unique among six species. A larger area of land for buildings increased the probability of distribution for *B. ardens* (Fig. S1(e)). In Japan, flower resources become scarce during the hot summer, and the abundance and species richness of bumblebees generally decrease in urban areas at low altitudes, except in the northern regions such as Hokkaido. However, *B. ardens* can even inhabit urban areas because they can build their nests between artificial structures (Iijima & Tamura 2003), and they produce gynes and males before the flower resources become scarce in the summer. Among bumblebee species in Japan, *B. ardens* is the earliest to begin its activity in spring, to end its activity in summer, and the probability increased with annual mean temperature (Fig. S2(b)). This might be related that the possible origin of *B. ardens* is the southern region in East Asia: the distribution of their related species *B. flavescens* covers the most southern regions among species in subgenus *Pyrobombus* (Cameron et al. 2007).

The percent contributions of temperature and altitude were high for *B. ignitus* (Table 1). However, for *B. ignitus*, these estimations were conducted using background data that included those from Hokkaido, which is not inhabited by *B. ignitus*. When we excluded Hokkaido from the bias file, the percent contributions of annual mean temperature and altitude were lower than those in the estimates obtained with Hokkaido (Table S3 in Appendix S3). The response curve shapes were almost the same for temperature and altitude (Figs. S2 and S3 in Appendixes S2 and S3), but we must carefully consider the effect of temperature and altitude on the probability of distribution for *B. ignitus*.

References

Cameron, S.A., Hines, H.M., Williams, P.H. 2007. A comprehensive phylogeny of the bumblebees (*Bombus*). Biological Journal of the Linnean Society, 91, 161-188.

Iijima, K., Tamura, M. 2003. House Insect Pests from the old nests of *Bombus ardens* Smith (Hymenoptera: Apidae), inside and outside artificial buildings. House and Household Pests, 24, 55-60 (in Japanese with English summary).


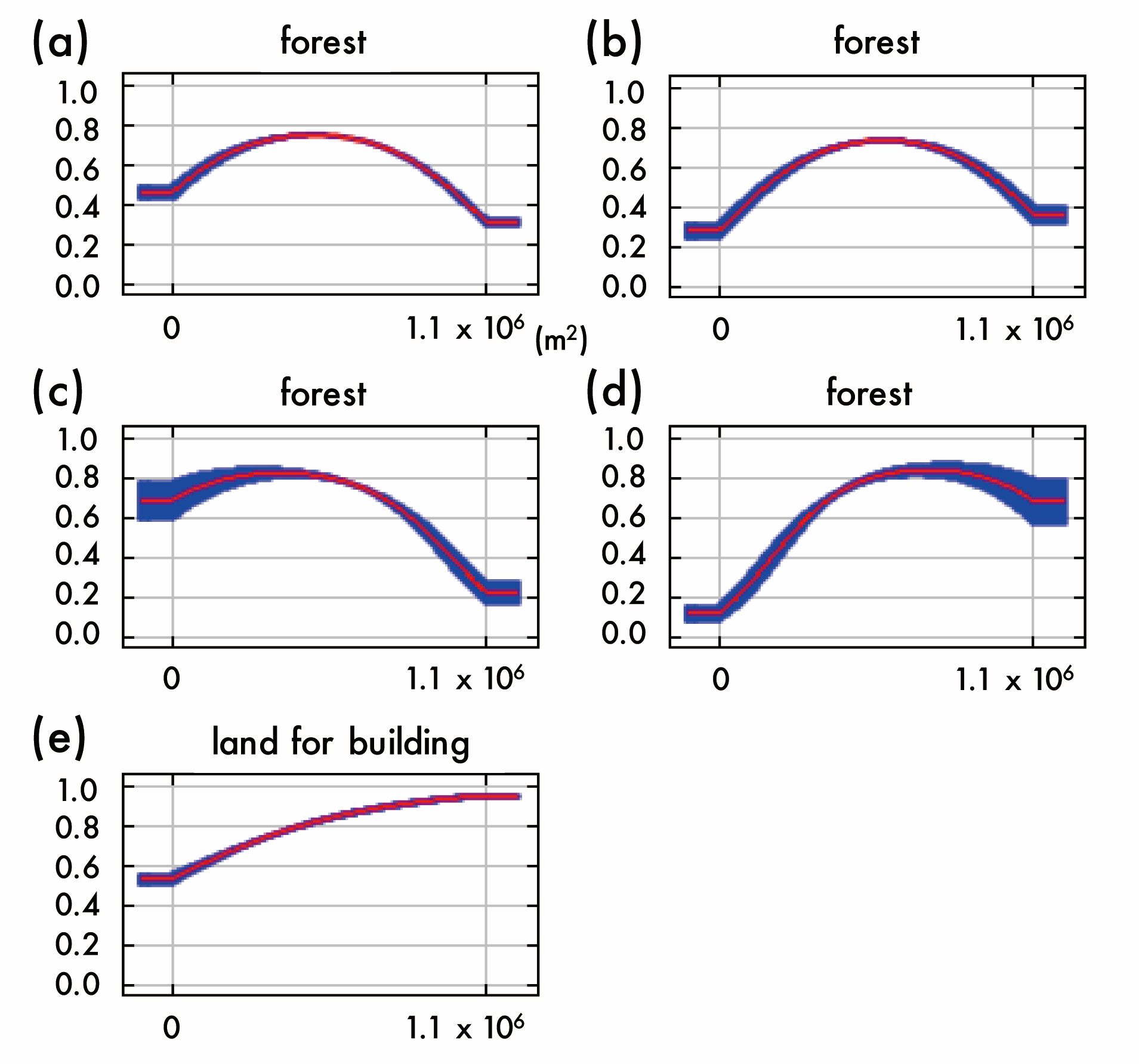


Figure S1. Marginal response curve of land use area for six bumble bee species using our citizen science data. (a) Forest area for *B. diversus*, (b) Forest area for *B. ardens*, (c) Forest area for *B. hypocrita*, (d) Forest area for *B. ignitus*, (e) Land area for buildings for *B. ardens*. Red curve indicates mean, and blue band indicates standard deviation.


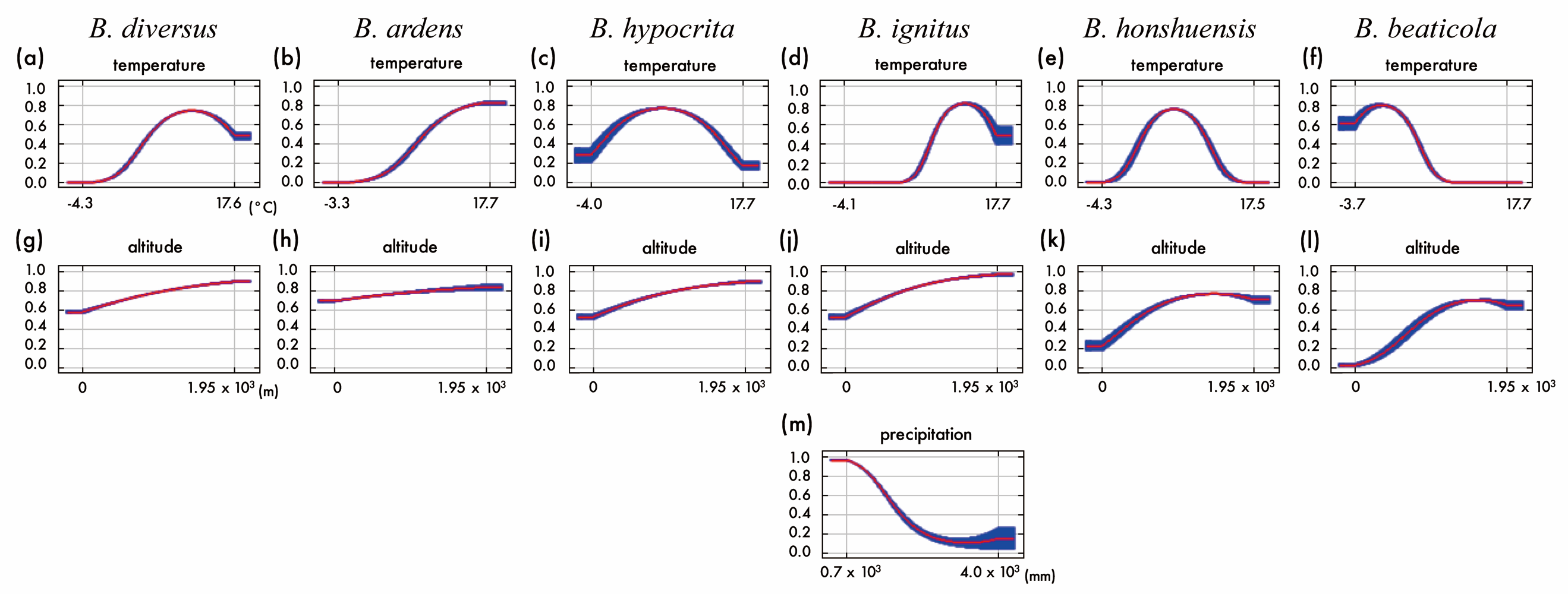


Figure S2. Marginal response curve of temperature, altitude, and precipitation for six bumble bee species using our citizen science data. (a) Temperature for *B. diversus*, (b) Temperature for *B. ardens*, (c) Temperature for *B. hypocrita*, (d) Temperature for *B. ignitus*, (e) Temperature for *B. honshuensis*, (f) Temperature for *B. beaticola*, (g) Altitude for *B. diversus*, (h) Altitude for *B. ardens*, (i) Altitude for *B. hypocrita*, (j) Altitude for *B. ignitus*, (k) Altitude for *B. honshuensis*, (l) Altitude for *B. beaticola*, and (m) Precipitation for *B. ignitus*. Red curve indicates the mean, and blue band indicates the standard deviation.

Appendix S3: Estimation of distributions using our citizen science data and background data without Hokkaido Prefecture.

In Japan, *B. ignitus* does not inhabit Hokkaido. There are two possible reasons why *B. ignitus* does not inhabit Hokkaido: (1) the environments in Hokkaido are unsuitable for *B. ignitus*, and (2) Tsugaru straits as a geographical barrier prevent *B. ignitus* from inhabiting Hokkaido. If it is attributed to only geographical barrier, using a bias file that includes Hokkaido may construct an inaccurate model. When a bias file that includes Hokkaido was used, the background data from Hokkaido that might have been suitable for *B. ignitus* were treated as unsuitable because there were no presence data for Hokkaido. Therefore, we used a bias file without Hokkaido to estimate the distribution for *B. ignitus*.

The Maxent estimates obtained using background data without Hokkaido showed that the distribution was determined mainly by forest areas and precipitation. The percent contributions of the annual mean temperature and altitude were lower whereas the percent contribution of precipitation was higher than those in the estimates obtained with Hokkaido (Table S3). The peak of the forest response curve shifted to a slightly smaller area (Fig. S3(a)), and the shape of the precipitation response curve became U-shaped curve (Fig. S3(b)), but the other response curves did not change greatly (Fig. S3(c) and (d)). The probability tended to be higher in the northern region of Japan, especially in some regions of Hokkaido (e.g., Sapporo, Ishikari, Otaru, and Iwanai-gun) (Figs. 4(d) and S4). In Japan, excluding Hokkaido, domesticated *B. ignitus* has been used as a pollinator of vegetables and fruits (e.g., tomato, and strawberry) cultivated in green houses. Thus, the risk of domesticated *B. ignitus* invading Hokkaido is high.

The other five species (*B. diversus*, *B. ardens*, *B. hypocrita*, *B. honshuensis* and *B. beaticola*) inhabit Hokkaido. Among these five species, the populations of four species (*B. diversus*, *B. ardens*, *B. hypocrita*, and *B. beaticola*) in Hokkaido are classified into separate subspecies (Table S1). Therefore, we also estimated the distribution for the species using a bias file and presence data without Hokkaido. The probability tended to be high in Hokkaido when we used a bias file and presence data without Hokkaido. The percent contributions of temperature for *B. ardens* and *B. diversus* decreased (23.2% to 9% for *B. ardens* and 14.5% to 7.6% for *B. diversus*) whereas those for *B. beaticola* increased (33% to 50.4% for *B. beaticola*). For *B. hypocrita,* the percent contribution of temperature was almost the same (12.7% to 12.3%). The percent contributions of altitude decreased for *B. beaticola*, but remained high (49.4% to 34.5% for *B. beaticola*). The percent contributions of altitude increased for *B. diversus* and *B. hypocrita* (21.3% to 26% for *B. diversus* and 32% to 42.9% for *B. hypocrita)*, but that for *B. ardens* was almost the same (1.2% to 1.6%). The percent contribution of temperature for *B. diversus* and *B. ardens* became relatively lower because the percent contribution of altitude for *B. diversus* and the percent contributions of forest areas for *B. diversus* and *B. ardens* all increased (altitude: 21.3% to 26% for *B. diversus*, forest: 30.7% to 42% for *B. diversus* and 12.7% to 24.3% for *B. ardens*). Therefore, we considered that the discriminating the subspecies of the four species was not necessary to estimate their distribution.

Table S3. Average percent contribution (Contribution) and permutation importance (Importance) of environmental factors in 100 Maxent estimates for *B. ignitus* using our citizen science data and the bias file without/with Hokkaido.

|  | without Hokkaido | | with Hokkaido | |
| --- | --- | --- | --- | --- |
|  | Contribution | Importance | Contribution | Importance |
| Altitude | 7.2 | 4 | 11 | 5.6 |
| Beach area | 0 | 0 | 0 | 0 |
| Body of seawater area | 0.3 | 0 | 0.5 | 0 |
| Forest area | 38 | 9.8 | 39.2 | 8.8 |
| Golf course area | 0.3 | 0.3 | 0.7 | 0.3 |
| Land for building area | 0.6 | 13 | 1.7 | 13 |
| Other agricultural land area | 2 | 3.3 | 3.5 | 4.8 |
| Other land area | 0.9 | 2.4 | 0.8 | 2.5 |
| Paddy field area | 1.8 | 4.4 | 0.5 | 6.2 |
| Precipitation | 33.2 | 30.9 | 15.3 | 16.1 |
| River and lakes area | 1.5 | 0.9 | 1 | 0.8 |
| Snowfall | 9.5 | 16 | 5.4 | 7.9 |
| Solar irradiance | 0.3 | 0.3 | 1.1 | 0.1 |
| Temperature | 3.2 | 14.6 | 16.8 | 33.7 |
| Wasteland area | 1.3 | 0.1 | 2.5 | 0.2 |


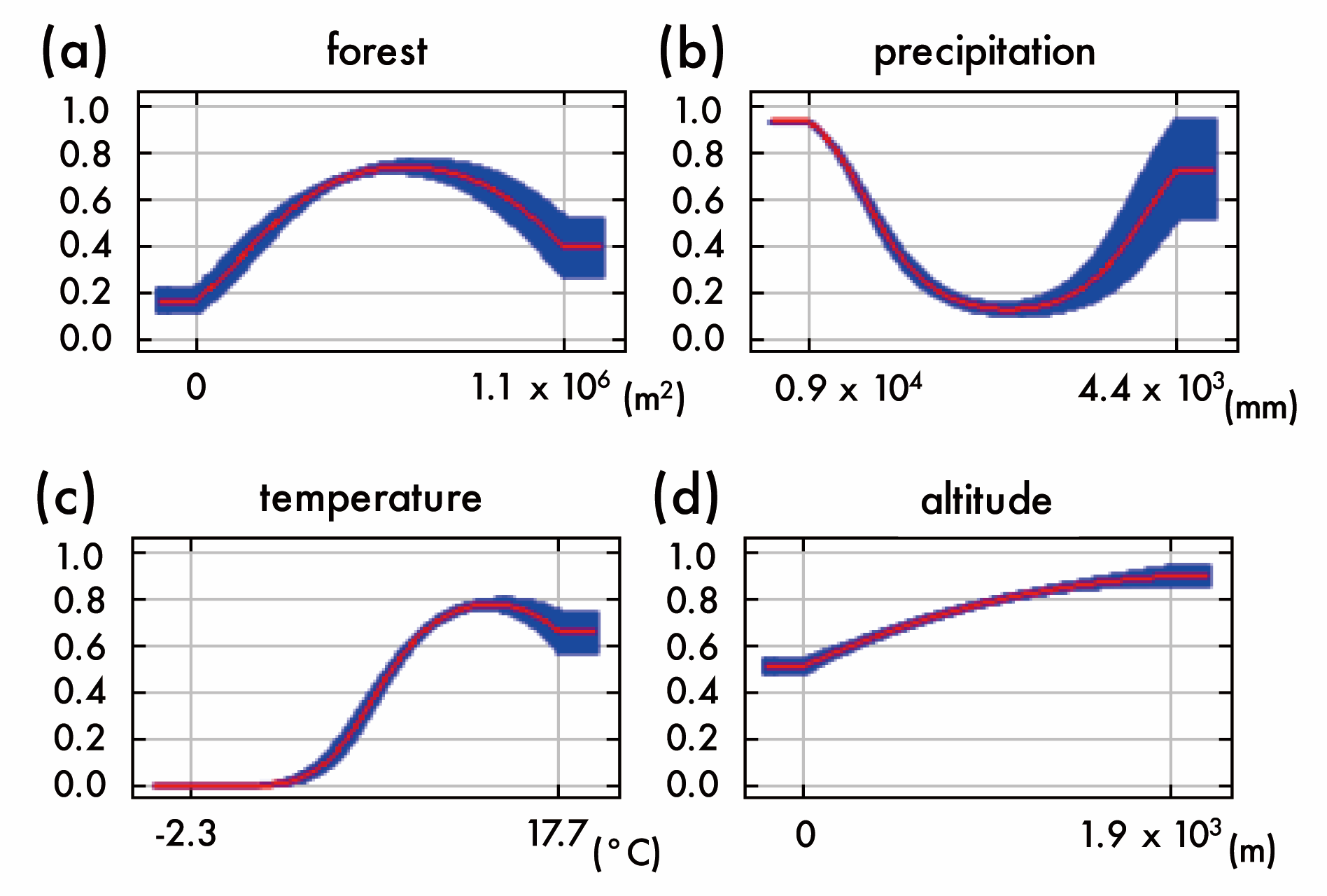


Figure S3. Marginal response curves of (a) forest area, (b) precipitation, (c) temperature, and (d) altitude in the estimates for *B. ignitus* using our citizen science data and the bias file without Hokkaido.


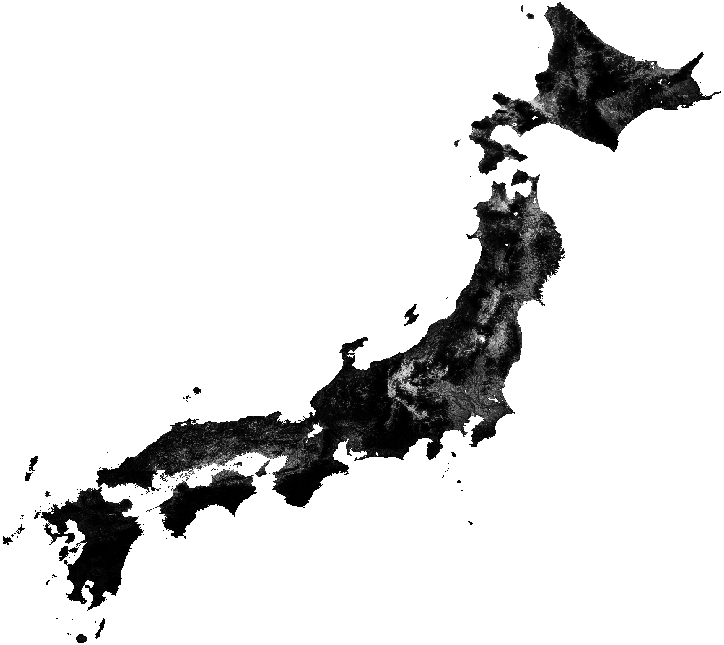


Figure S4. Prediction obtained using our citizen science data and the bias file without Hokkaido. This map also indicates the probability of domesticated *B. ignitus*'s invading Hokkaido. This map was drawn with the software ArcGIS ver. 10.0 (https://www.arcgis.com/features/index.html).

Appendix S4. The effects of land use types, temperature, and altitude on bumblebee distributions estimated using GBIF data

The standard deviations of ROC were greater than those of our citizen science data (Fig. S5). The percent contributions of land for building areas were high for *B. diversus*, *B. ardens*, *B. hypocrita*, and *B. honshuensis* (Table 2). The marginal response curves of land for building area for these species were increasing function versus the area (Fig. S6 (a), (b), (c), and (d)). The percent contribution of forest area was the secondary largest for *B. hypocrita* (Table 2), but the marginal response curve of forest area for *B. hypocrita* (Fig. S5(e)) was different from that of our citizen science data (Fig. S1(c)).

The percent contribution of temperature was high whereas that of altitude was low for five species (Table 2). The marginal response curve of temperature for four species was an increasing function (Fig. S7(a), (b), (c), and (d)), and that for *B. honshuensis* was almost constant (Fig. S7(e)). The standard deviations of marginal response curves of altitude were large for all five species (Fig. S7(f), (g), (h), (i), and (j)). The percent contribution of global solar irradiance for *B. ignitus* was high, and a decreasing function versus it (Fig. S7(k)).


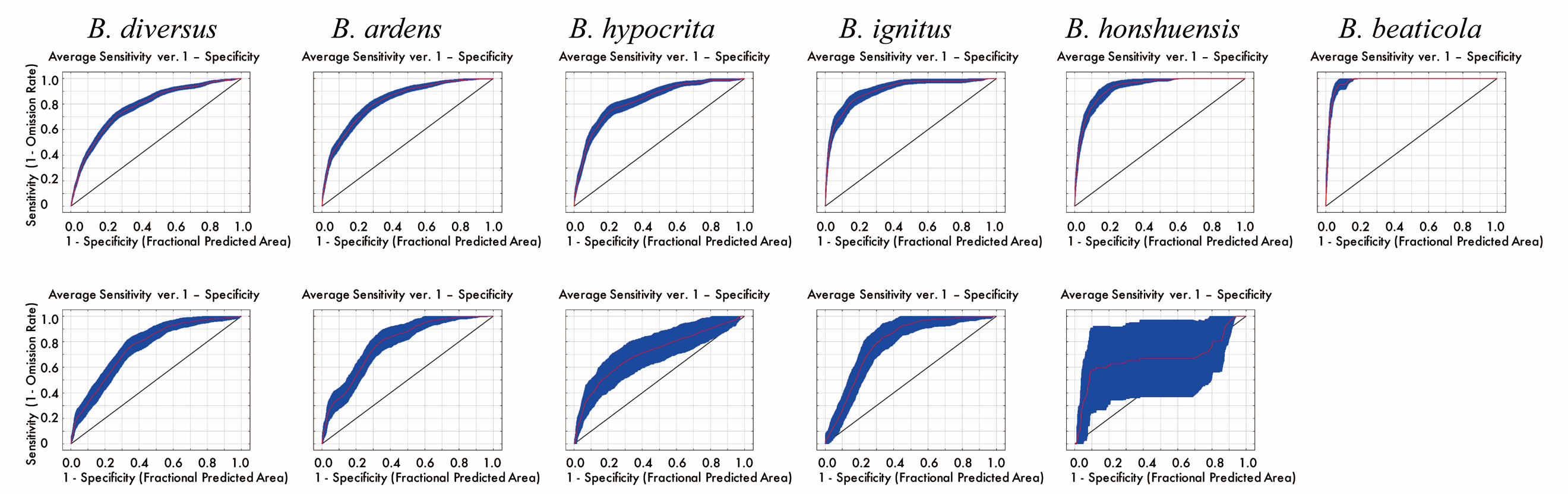


Figure S5. ROC curves of our citizen science and GBIF data. Upper graphs are those of our citizen science data, and lower graphs are those of GBIF data. Red curve indicates the mean, and blue band indicates the standard deviation.


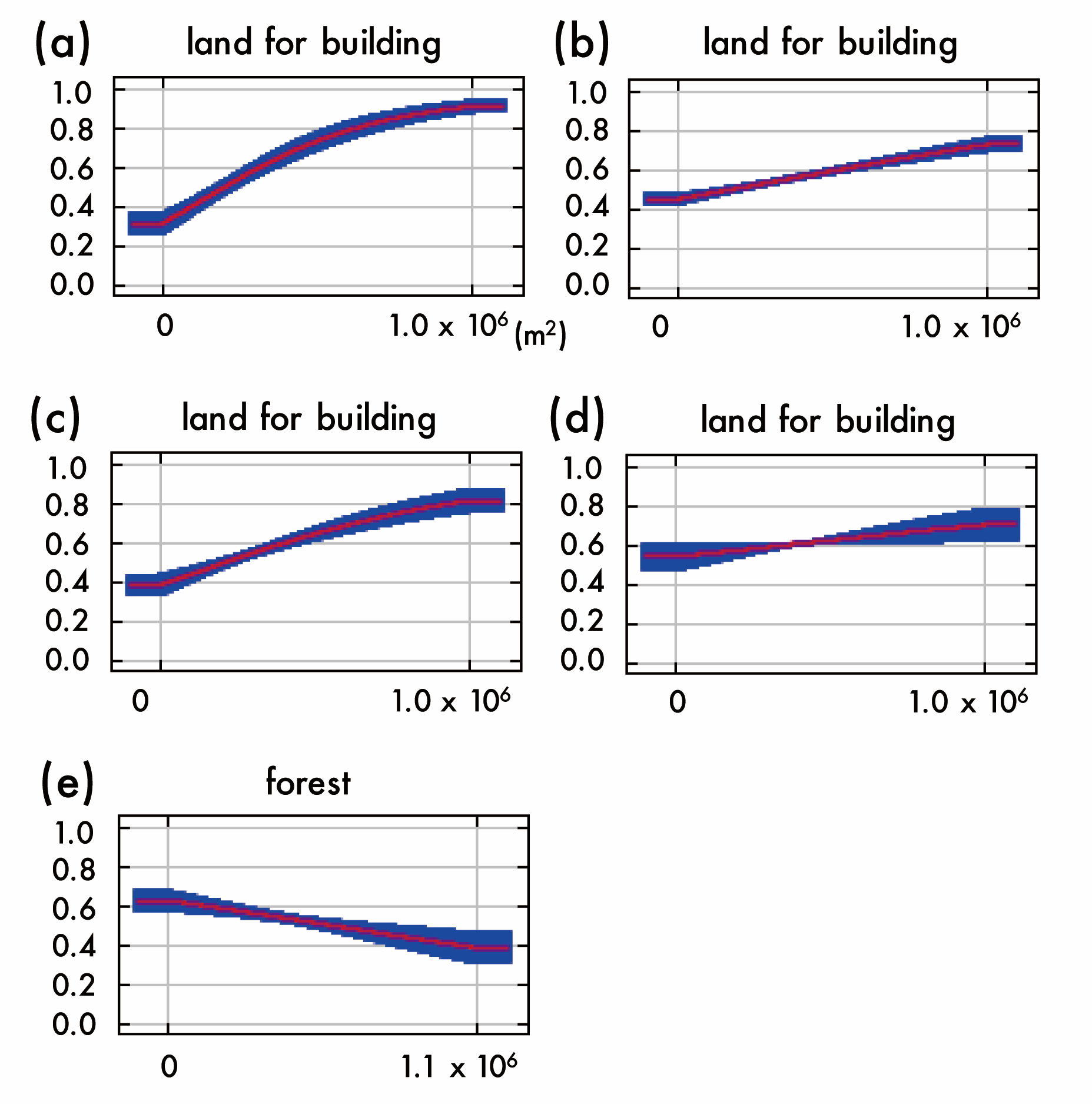


Figure S6. Marginal response curves of land use areas in the estimates using GBIF data. (a) land for building area for *B. diversus*, (b) land for building area for *B. ardens*, (c) land for building for *B. hypocrita*, (d) land for building for *B. honshuensis*, and (e) forest area for *B. hypocrita*. Red curve indicates the mean, and blue band indicates the standard deviation.


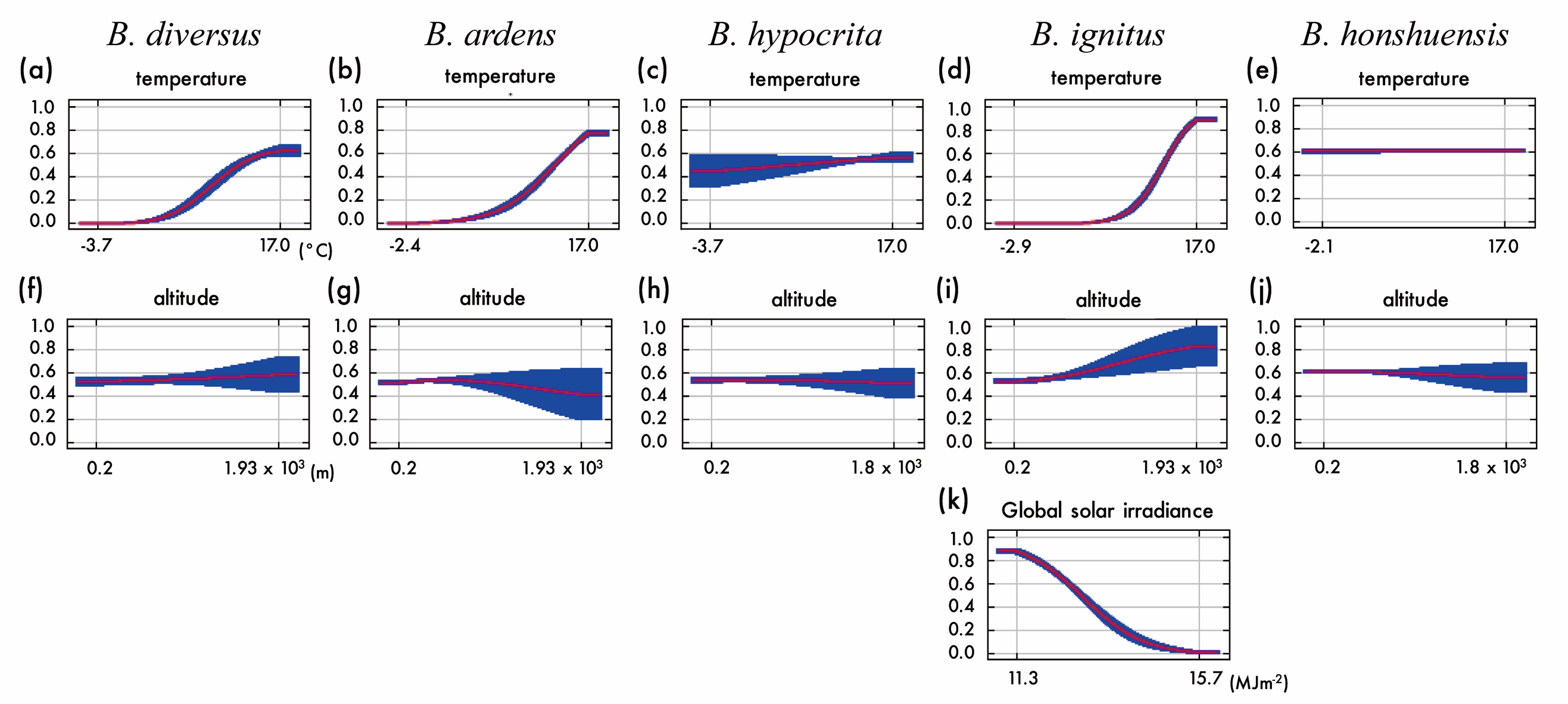


Figure S7. Marginal response curves of temperature, altitude, and global solar irradiance in the estimates using GBIF data. (a) Temperature for *B. diversus*, (b) Temperature for *B. ardens*, (c) Temperature for *B. hypocrita*, (d) Temperature for *B. ignitus*, (e) Temperature for *B. honshuensis*, (f) Altitude for *B. diversus*, (g) Altitude for *B. ardens*, (h) Altitude for *B. hypocrita*, (i) Altitude for *B. ignitus*, (j) Altitude for *B. honshuensis*, and (k) Global solar irradiance for *B. ignitus*. Red curve indicates the mean, and blue band indicates the standard deviation.

Appendix S5.

Table S4. Environmental variables used in Maxent.

| Environmental data (year) | Variables |
| --- | --- |
| Land use area data (2014) | Paddy field |
|  | Other agricultural land (crops and orchards) |
|  | Forest |
|  | Wasteland |
|  | Land for building |
|  | Other land (e.g., artificial land) |
|  | Rivers and lakes |
|  | Beach |
|  | Body of seawater |
|  | Golf course |
| Climate data (1981-2010) | Annual precipitation |
|  | Annual mean temperature |
|  | Maximum snowfall |
|  | Mean of total global solar irradiance |
| Altitude and slope data (2011) | Mean altitude |
